# Imaging findings in COVID-19 pneumonia

**DOI:** 10.6061/clinics/2020/e2027

**Published:** 2020-06-16

**Authors:** Lucas de Pádua Gomes de Farias, Eduardo Kaiser Ururahy Nunes Fonseca, Daniel Giunchetti Strabelli, Bruna Melo Coelho Loureiro, Yuri Costa Sarno Neves, Thiago Potrich Rodrigues, Rodrigo Caruso Chate, Cesar Higa Nomura, Márcio Valente Yamada Sawamura, Giovanni Guido Cerri

**Affiliations:** I Instituto de Radiologia (InRad), Hospital das Clinicas HCFMUSP, Faculdade de Medicina, Universidade de Sao Paulo, Sao Paulo, SP, BR; II Instituto do Coracao (InCor), Hospital das Clinicas (HCFMUSP), Faculdade de Medicina, Universidade de Sao Paulo, Sao Paulo, SP, BR; III Instituto do Cancer do Estado de São Paulo (ICESP), Hospital das Clinicas (HCFMUSP), Faculdade de Medicina, Universidade de Sao Paulo, Sao Paulo, SP, BR

**Keywords:** SARS-CoV-2, COVID-19, Coronavirus, Radiography, Computed Tomography, Ultrasonography

## Abstract

The coronavirus disease (COVID-19), caused by the severe acute respiratory syndrome coronavirus 2 (SARS-CoV-2), emerged in Wuhan city and was declared a pandemic in March 2020. Although the virus is not restricted to the lung parenchyma, the use of chest imaging in COVID-19 can be especially useful for patients with moderate to severe symptoms or comorbidities. This article aimed to demonstrate the chest imaging findings of COVID-19 on different modalities: chest radiography, computed tomography, and ultrasonography. In addition, it intended to review recommendations on imaging assessment of COVID-19 and to discuss the use of a structured chest computed tomography report. Chest radiography, despite being a low-cost and easily available method, has low sensitivity for screening patients. It can be useful in monitoring hospitalized patients, especially for the evaluation of complications such as pneumothorax and pleural effusion. Chest computed tomography, despite being highly sensitive, has a low specificity, and hence cannot replace the reference diagnostic test (reverse transcription polymerase chain reaction). To facilitate the confection and reduce the variability of radiological reports, some standardizations with structured reports have been proposed. Among the available classifications, it is possible to divide the radiological findings into typical, indeterminate, atypical, and negative findings. The structured report can also contain an estimate of the extent of lung involvement (e.g., more or less than 50% of the lung parenchyma). Pulmonary ultrasonography can also be an auxiliary method, especially for monitoring hospitalized patients in intensive care units, where transfer to a tomography scanner is difficult.

## INTRODUCTION

In December 2019, an outbreak of a highly contagious pneumonia of unknown etiology was reported in the city of Wuhan, China, with many infected patients presenting severe acute respiratory syndrome (SARS). It quickly spread to other countries and was declared a pandemic in March 2020 by the World Health Organization (1,2). The etiological agent, identified from epithelial cells of infected patients’ airways, was a coronavirus (SARS-CoV-2), belonging to subgenus Sarbecovirus and subfamily Orthocoronavirinae, the seventh member of the coronavirus family that is known to infect humans (1). The infection was named coronavirus disease (COVID-19).

COVID-19 pneumonia shares etiological and clinical similarities to other contemporary syndromes also caused by coronaviruses, including the Middle East Respiratory Syndrome (MERS), identified in 2012, and SARS, in 2003 (3). Similar to other viral infectious diseases, COVID-19 is not restricted to the pulmonary parenchyma, with reports of myocarditis, hypercoagulability status, acute renal failure, mesenteric lymphadenitis, and encephalitis (4).

This article aimed to demonstrate the chest imaging findings of COVID-19 on different modalities, to review national and international recommendations on imaging assessment of COVID-19 (5-9), and to discuss the use of a structured chest computed tomography (CT) report for the disease.

## ROLE OF IMAGING IN COVID-19 PULMONARY INFECTION

Chest imaging should be carefully indicated in patients with suspected COVID-19 infection not only to reduce the patients radiation exposure but also to reduce unnecessary exposure of other patients and healthcare workers, and to rationalize the use of personal protective equipment and resources for disinfecting the patient care equipment (9).

**Figure 1:**
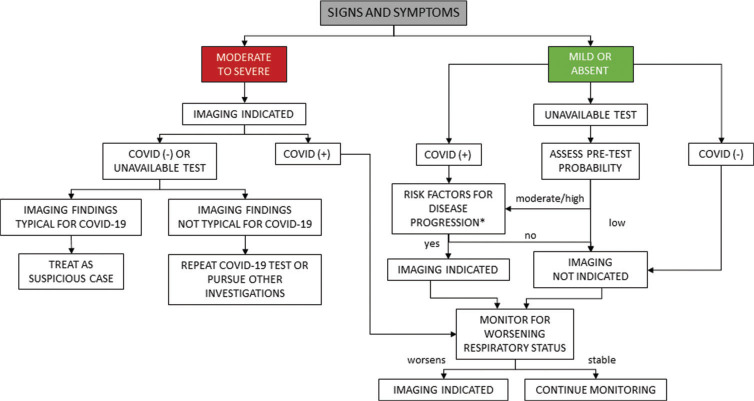
Recommendations for performing imaging in patients with COVID-19 pneumonia. Adapted from Rubin et al (9). * Age >65 years, cardiovascular diseases, hypertension, chronic respiratory diseases, diabetes, and immunosuppression.

The use of chest imaging in COVID-19 suspected cases does not replace specific diagnostic tests such as the detection of viral RNA by reverse transcription polymerase chain reaction (RT-PCR) and serological detection of antibodies to SARS-CoV-2. Moreover, most medical societies do not recommend the use of imaging as a method of disease screening (5-8,10). In general, it is not indicated for asymptomatic patients or those with mild symptoms of the disease. Imaging should be reserved for those with moderate to severe symptoms, those with risk of progression (presence of comorbidities), and those with worsening of the respiratory condition (Figure 1). In environments with limited resources, imaging can eventually be indicated as a method for medical triage of patients with moderate to severe clinical features and a high pre-test probability (9), in whom urgent decision-making is of primary importance.

Notably, there is an overlap of chest imaging findings in COVID-19 and other diseases (8). In addition, pulmonary imaging features can persist for weeks to months and should not be an objection factor for patient discharge (5), nor should it be considered as a treatment control method (6). In general, the resolution of the imaging findings is observed at approximately the 26^th^ day of symptom onset in patients with COVID-19 pneumonia (6), but in some cases, it can take even longer.

## CHEST RADIOGRAPHY

Chest radiography is a quick and easy method, frequently requested due to its wide availability and low cost. The advent of portable devices has allowed its use in intensive care units and field hospitals (7).

**Figure 2:**
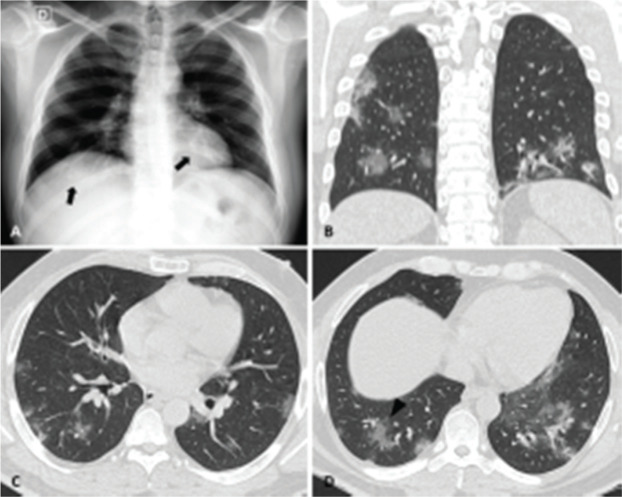
Chest radiography (A), posteroanterior view, shows retrocardiac and right peri diaphragmatic opacities (arrow), regions which are often neglected in the radiographic evaluation; there are no other notable parenchymal changes. Coronal (B) and axial (C and D) CT images on the same day show ground glass opacities associated with some consolidations in the posterior regions of both lungs, most prominent in the lower lobes. Note the slight thickening of the inter and intralobular septa that constitute the crazy-paving pattern in the right lung base (arrowhead).

**Figure 3:**
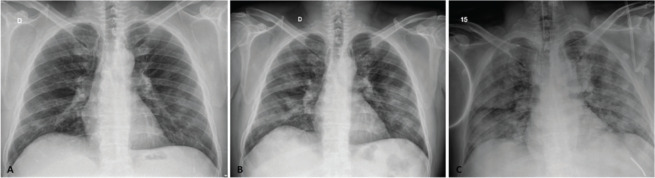
Chest radiographs, posteroanterior views (A and B) and in bed (C), performed at intervals of 2 days, show progression of the diffuse and bilateral pulmonary opacities. Note the tracheal cannula and other vital devices (C).

Radiologists and clinicians should be aware of the radiography limitation of COVID-19 pneumonia due to the low sensitivity, estimated at 25% (11), especially in initial cases (Figure 2). Therefore, it should not be considered as a screening method (7). It is recommended for selected populations, such as hospitalized patients to assess disease progression (Figure 3) or to assess associated complications, such as ventilator-associated pneumonia, pleural effusion, or pneumothorax (9).

The main radiographic findings are lung opacities with bilateral distribution and predominance in the periphery and lower pulmonary fields (12). The disease extension can be quantified utilizing adapted scores according to the percentage of pulmonary opacity extension (12). It is noteworthy that such information can be either under-detected or underestimated when compared to CT, thus limiting the disease monitoring capability of radiography (11).

## CHEST COMPUTED TOMOGRAPHY

In the context of the COVID-19 pandemic, the use of CT has increased significantly, despite the aforementioned national and international recommendations. It should be performed in hospitalized and symptomatic patients with clinical worsening and/or patients who have comorbidities (5-8,10). In these patients, CT is indicated mainly to assess the extent of the disease and to identify complications, such as pulmonary thromboembolism or overlapping bacterial infection, and to evaluate differential diagnoses (6).

When compared to the reference diagnostic test (RT-PCR), CT has high sensitivity (97%), but low specificity (25%) (13), and hence cannot replace it (7). The tomographic findings considered typical of COVID-19 are ground glass opacities (GGOs), consolidations and crazy-paving pattern (Figure 2) with bilateral and multifocal distribution, and a peripheral and posterior predominance (14-16). Such findings are not exclusive to COVID-19 and can be found in other viral pneumonias, connective tissue lung disease, and drug-induced lung disease (8). Therefore, clinical data and temporal correlation with radiological findings are extremely important, as well as correlation with previous examination findings. In addition to these findings, CT of patients with COVID-19 may also show signs of organizing pneumonia, reversed halo sign, reticular pattern, subpleural curvilinear lines, parenchymal bands, pseudocavities, and nodules, sometimes configuring the halo sign (17-19). Airway centered disease such as bronchial wall thickening, centrilobular and tree in bud opacities, pleural effusion, and lymphadenopathy are not frequently encountered at the initial presentation of COVID-19 (17).

CT findings also vary according to the days of symptom onset. GGOs are more common in the first few days of symptom onset and can evolve to consolidations, reaching a peak in pulmonary opacities between the 9th and 13^th^ days after symptoms onset (6,15).

The dissociation between laboratory and tomographic findings may be seen in patients with positive RT-PCR and absence of imaging findings and those with extensive imaging findings and negative RT-PCR (7,20).

## CT STRUCTURED REPORT IN COVID-19

**Figure 4:**
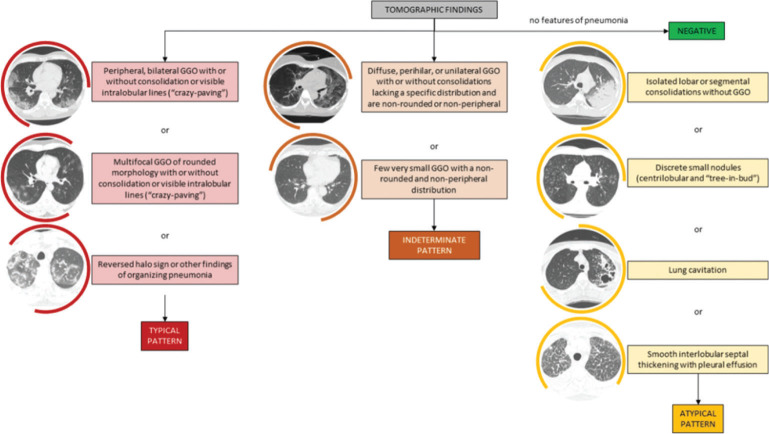
Recommendations for categorizing computed tomography findings of COVID-19 pneumonia. Adapted from Simpson et al (8).

During a pandemic, a more assertive interpretation of CT findings related to COVID-19 has become necessary to assist patient management. Therefore, some groups (8,21) have proposed the elaboration of structured reports. This type of report offers advantages in the context of high demand of examinations and the need for prompt decisions. For the radiologist, it helps in the elaboration of the report, decrease the reporting time and variability, and reduce uncertainty in reporting findings. For the referring physician, it improves the understanding of the radiological findings and the quality of the information transmitted, allowing better clinical management (22). Among available classifications, the proposal by the consensus of specialists of the Radiological Society of North America (8) has been widely used. They propose four groups of CT findings: typical, indeterminate, atypical, and negative for COVID-19 (Figure 4) and suggest ways to report them. In our hospital, we adapted this classification, with small reporting language changes (Table 1).

**Figure 5:**
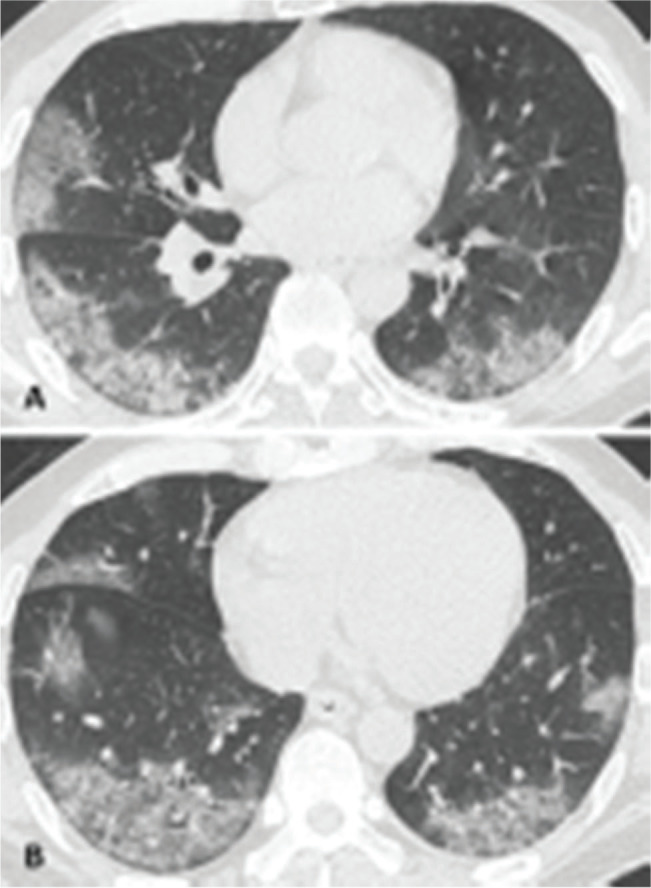
Typical findings of COVID-19. Axial CT images show peripheral, bilateral GGOs with areas of consolidation. Note the associated septal thickening.

**Figure 6:**
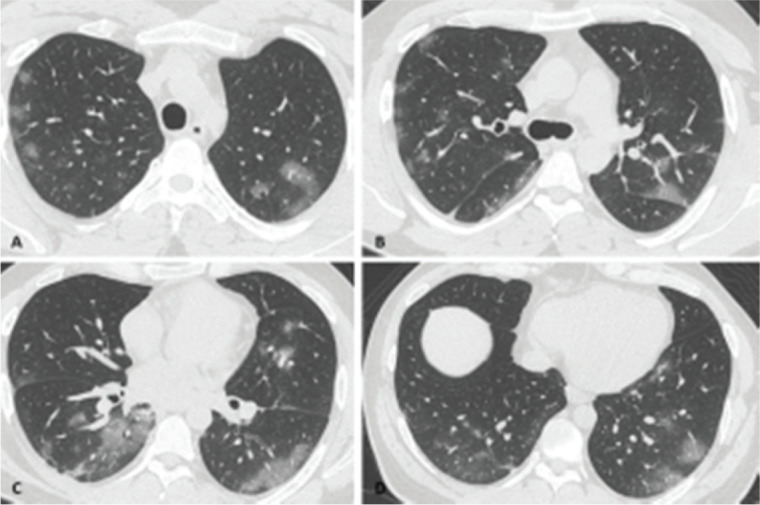
Typical findings of COVID-19. Axial CT images show multifocal GGOs of rounded morphology with areas of consolidation.

**Figure 7:**
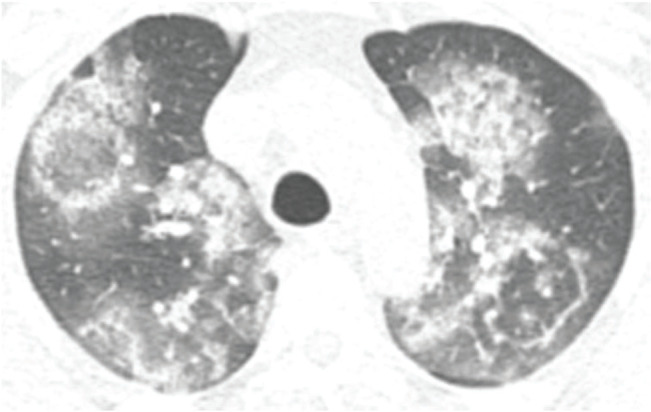
Typical findings of COVID-19. Axial CT image shows GGO areas surrounded by rings of consolidation (reversed halo sign), complete and incomplete.

The typical CT finding for COVID-19 consists of (1) peripheral, bilateral GGO with or without consolidation or visible intralobular lines (crazy-paving pattern) (Figure 5); (2) multifocal GGO of rounded morphology with or without consolidation or visible intralobular lines (crazy-paving pattern) (Figure 6); or (3) reversed halo sign or other findings of organizing pneumonia (Figure 7). These findings are suggestive of pneumonia and viral etiology must be included in the etiological differential diagnosis, particularly COVID-19.

**Figure 8:**
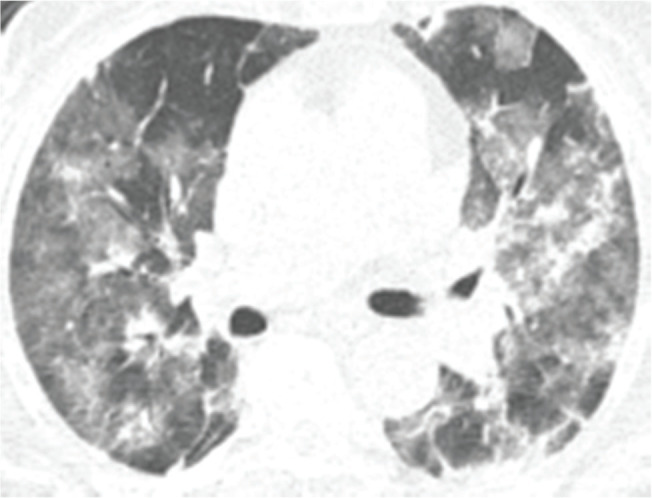
Indeterminate findings of COVID-19. Axial CT image shows bilateral diffused GGOs associated with consolidations, and some areas of septal thickening.

**Figure 9:**
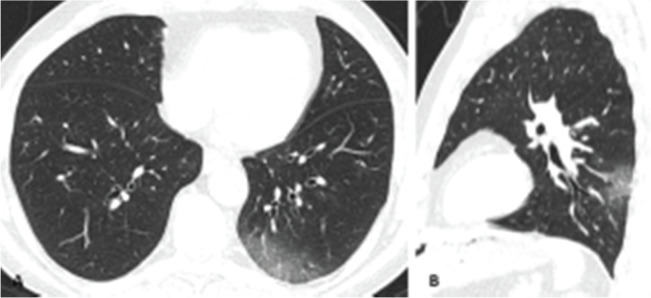
Indeterminate findings of COVID-19. Axial (A) and sagittal (B) CT images show unilateral GGOs in the upper segment of the left lower lobe.

**Figure 10:**
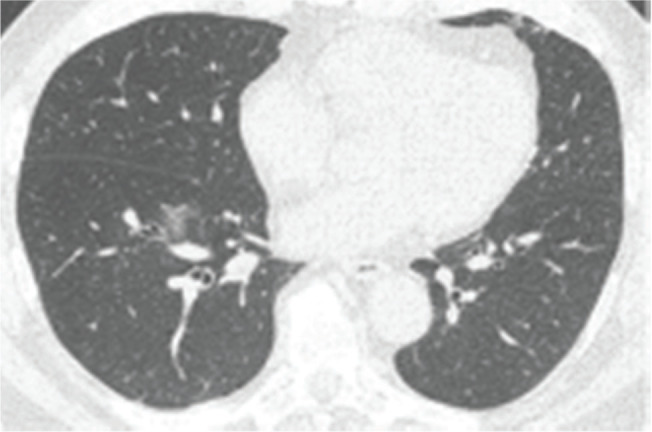
Indeterminate findings of COVID-19. Axial CT image shows a very small GGO with a non-rounded and non-peripheral distribution.

The indeterminate CT appearance for COVID-19 consists of diffuse (Figure 8), perihilar, or unilateral (Figure 9) GGO with or without consolidation lacking a specific distribution. Patients with few very small GGOs with a non-rounded and non-peripheral distribution (Figure 10) are also included in this group. In these cases, the imaging findings are nonspecific and can be observed in several diseases of non-infectious and infectious origin, including viral pneumonia. Although these are not a suggestive tomographic appearance, the possibility of COVID-19 should not be ruled out, especially in severe clinical cases with extensive pulmonary opacities, or in mild cases with isolated pulmonary opacities.

**Figure 11:**
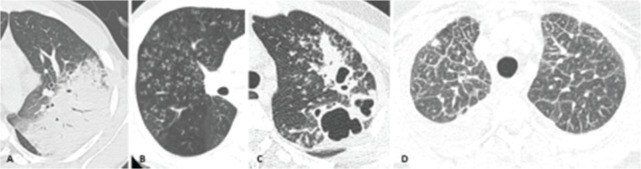
Atypical findings of COVID-19. Axial CT images show (A) isolated segmental consolidation; (B) discrete small centrilobular nodules, some of them with the “tree-in-bud” pattern; (C) lung cavitations; and (D) bilateral smooth interlobular septal thickening with pleural effusion.

Patients with pulmonary infection findings different from those previously described above can be categorized into the atypical appearance (Figure 11), such as isolated lobar or segmental consolidations, discrete small nodules (centrilobular and tree-in-bud appearance), lung cavitation, and smooth interlobular septal thickening with pleural effusion (8). In this group, imaging findings are not usually reported in COVID-19 infection, and other etiological agents should be considered initially.

The last group is that of patients without features suggestive of pneumonia. As already discussed, a normal chest CT does not exclude the diagnosis of COVID-19, especially in the initial days of symptoms onset.

In addition to this classification, the structured report may also contain an estimate of the extent of pulmonary involvement (e.g., describing whether it involves more or less than 50% of the lung parenchyma), information that has been used as one of the criteria for hospitalization (23), and is also related to admission to intensive care units and mortality (24,25). Such quantification may be performed visually or automatically through specific software. Several imaging scores (by lobes or pulmonary fields) have been proposed (14,23,26,27), but all of them are difficult to apply in clinical practice and more studies are needed to validate the correlation of these quantification methods with the clinical course of the disease.

## ULTRASONOGRAPHY

As previously mentioned, COVID-19 alterations tend to predominate in the lung periphery, which makes lung ultrasound an option in the follow-up of hospitalized patients, especially those in intensive care units, where transfer to a CT scanner is impossible or difficult. However, this method should not be used as a substitute for CT (5,6,28).

**Figure 12:**
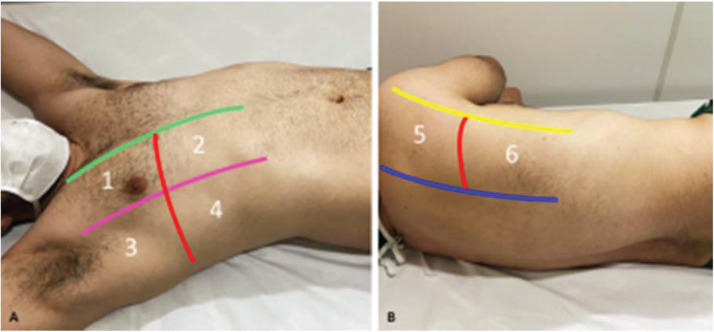
Transducer sites for lung ultrasound. The six sites are anatomically divided by the parasternal line (green line), anterior axillary line (pink line), posterior axillary line (yellow line), and paravertebral line. The subdivision in the superior and inferior takes into account the well-established Bedside Lung Ultrasound in Emergency (BLUE) protocol.

**Figure 13:**
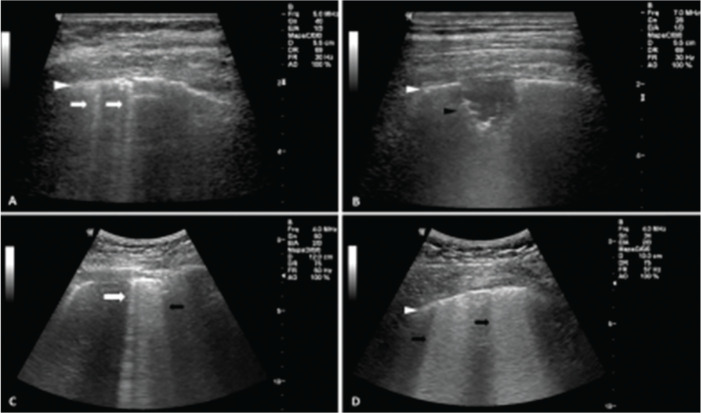
Lung ultrasound. Linear (A and B) and convex (C and D) transducers in intercostal spaces show irregular and thickened pleural line (white arrowhead); hypoechogenic image with irregular contours, compatible with subpleural consolidation (black arrowhead); multifocal B-lines (white arrow), some of which are coalescent (black arrow), characterizing the appearance of a white lung on ultrasound (A).

Its potential use as a serial assessment tool through established protocols, such as the 12-site scanning technique (29,30) (Figure 12), allows the evaluation of the progression of inflammatory involvement of the parenchyma and identification of mild findings, such as pleural thickening and even small subpleural consolidations (Figure 13). It also reduces environmental exposure as only a single doctor is needed to perform the examination at bedside (5,28).

Lung ultrasound is based on artefactual lines and their patterns. In patients with COVID-19, there are irregularly spaced vertical artifacts, known as B-lines, which may coalesce. Consolidations with or without air bronchograms may also be found in these patients (28) (Figure 13). B-lines represent the interlobular and/or intralobular septal thickening, most associated with pulmonary edema and interstitial disorders. When coalescent, with several B-lines grouped, they correspond to GGO at the periphery of the lungs, as observed on CT (31,32).

## CONCLUSION

Imaging should be wisely indicated in the evaluation of patients with COVID-19 to avoid unnecessary exposure of other patients and healthcare workers and rationalize the use of personal protective equipment. Imaging should be reserved for patients with moderate to severe respiratory symptoms, risk of progression (presence of comorbidities), or worsening of the respiratory condition. It should not be indicated for asymptomatic patients or those with mild symptoms. In environments with limited testing resources, imaging can eventually be indicated as a triage method for suspected patients with high pre-test probability.

Chest CT is the main imaging method used in the evaluation of COVID-19 pneumonia. Typical findings include GGOs with or without consolidation, crazy-paving pattern with bilateral and multifocal distribution, peripheral and posterior predominance, multifocal GGOs of rounded morphology, and reversed halo sign. Additionally, CT can help evaluate the extent of pulmonary disease, presence of complications, and differential diagnosis. Despite typical CT findings in COVID-19, RT-PCR remains the gold standard for the diagnosis.

Structured chest CT report standardizes the imaging findings and optimizes communication with the referring physician, thus being a useful tool in the pandemic scenario.

## AUTHOR CONTRIBUTIONS

Farias LPG, Fonseca EKUN, Strabelli DG, Loureiro BMC, Neves YCS, and Rodrigues TP were responsible for the study conceptualization and manuscript drafting. Chate RC, Nomura CH and Sawamura MVY were responsible for the manuscript review. Cerri GG was responsible for manuscript review and supervision.

## Figures and Tables

**Table 1:** Adapted Proposed Reporting Language for CT Findings Related to COVID-19 used in the Hospital das Clínicas da Faculdade de Medicina da USP.

Appearance	Suggested reporting language
Typical	Imaging features are suggestive of pneumonia and viral etiology must be included in the etiological differential diagnosis, particularly COVID-19.
Indeterminate	Imaging features are nonspecific and can be found in a variety of infectious and non-infectious processes, including cases of viral pneumonia and COVID-19.
Atypical	Imaging features suggest pulmonary inflammation/infection, although its pattern is not usually reported in COVID-19 cases. Other etiological agents should be considered initially.
Negative for pneumonia	No CT findings suggestive of pulmonary inflammation/infection. Note: CT may be negative in some early stages of COVID-19.
